# New Insights into the Type II Toxins from the Sea Anemone *Heteractis crispa*

**DOI:** 10.3390/toxins12010044

**Published:** 2020-01-10

**Authors:** Rimma S. Kalina, Steve Peigneur, Elena A. Zelepuga, Pavel S. Dmitrenok, Aleksandra N. Kvetkina, Natalia Y. Kim, Elena V. Leychenko, Jan Tytgat, Emma P. Kozlovskaya, Margarita M. Monastyrnaya, Irina N. Gladkikh

**Affiliations:** 1 G.B. Elyakov Pacific Institute of Bioorganic Chemistry, Far Eastern Branch of the Russian Academy of Science, 690022 Vladivostok, Russia; zel@piboc.dvo.ru (E.A.Z.); paveldmt@piboc.dvo.ru (P.S.D.); kvetkinaan@gmail.com (A.N.K.); natalya_kim@mail.ru (N.Y.K.); leychenko@gmail.com (E.V.L.); kozempa@mail.ru (E.P.K.); rita1950@mail.ru (M.M.M.); 2 Toxicology and Pharmacology, University of Leuven (KU Leuven), Campus Gasthuisberg, O&N 2, Herestraat~49, P.O. Box 922, 3000 Leuven, Belgium

**Keywords:** sea anemone, type II toxins, voltage-gated sodium channels, electrophysiology

## Abstract

Toxins modulating Na_V_ channels are the most abundant and studied peptide components of sea anemone venom. Three type-II toxins, δ-SHTX-Hcr1f (= RpII), RTX-III, and RTX-VI, were isolated from the sea anemone *Heteractis crispa*. RTX-VI has been found to be an unusual analog of RTX-III. The electrophysiological effects of *Heteractis* toxins on nine Na_V_ subtypes were investigated for the first time. *Heteractis* toxins mainly affect the inactivation of the mammalian Na_V_ channels expressed in the central nervous system (Na_V_1.1–Na_V_1.3, Na_V_1.6) as well as insect and arachnid channels (BgNa_V_1, VdNa_V_1). The absence of Arg13 in the RTX-VI structure does not prevent toxin binding with the channel but it has changed its pharmacological profile and potency. According to computer modeling data, the δ-SHTX-Hcr1f binds within the extracellular region of the rNa_V_1.2 voltage-sensing domain IV and pore-forming domain I through a network of strong interactions, and an additional fixation of the toxin at the channel binding site is carried out through the phospholipid environment. Our data suggest that *Heteractis* toxins could be used as molecular tools for Na_V_ channel studies or insecticides rather than as pharmacological agents.

## 1. Introduction

Voltage-gated sodium channels (Na_V_) are key elements for the transmission of electrical signals, as they initiate and propagate action potentials in excitable neuronal, cardiac, and skeletal muscle cells. Na_V_ channels also have non-canonical functions in non-excitable cells [1,2]. The electrophysiological properties of Na_V_ channels and their topology have been studied well, but many questions concerning the role of certain Na_V_ subtypes in an organism, details of the structure, and the mechanisms of channel interaction with various modulators are currently under consideration.

Eukaryotic Na_V_ channels are transmembrane protein complexes consisting of a highly conserved pore-forming α-subunit (260 kDa) having four homologous domains and 1–4 auxiliary β-subunits (30–40 kDa) intrinsic for vertebrates. Each domain is composed of six transmembrane segments, with the fourth segment acting as a voltage sensor [3]. Na_V_1.1–Na_V_1.3 and Na_V_1.6 are the main channels of the central nervous system (CNS). Na_V_1.7–Na_V_1.9 are mostly expressed in the peripheral nervous system (PNS). Electroexcitability of the cardiac and skeletal muscle cells involves Na_V_1.5 and Na_V_1.4 channels, respectively. In accordance with Na_V_ localization in an organism, most of the diseases associated with Na_V_ channelopathies affect the nervous, cardio-vascular, or musculoskeletal systems [1,2].

Known modulators of Na_V_ vary in terms of chemical nature and binding sites (pore-forming or voltage-sensitive domains). To date, a least eight binding sites localized on the α-subunits of the channel have been identified. At the same time, the regulatory β-subunits can influence the affinity and activity of toxins towards Na_V_ channels [4].

Na_V_ sea anemone toxins (3–5 kDa) are the most abundant peptide components of sea anemone venom. At present, more than fifty toxins have been isolated from various sea anemone species [5]. Based on the identity of the amino acid sequences, they are classified into four structural types. The type I and II toxins include 45–54 aa (amino acid residues) and are believed to have the same ancestor gene [6,7]. The amino acid sequence identity between toxins of these types is within 50%. Toxicity in both mammals and crustaceans has been shown for the type I and II toxins [8,9,10]. 

The vast majority of experiments elucidating the effects of sea anemone toxins on Na_V_ currents were carried out using the type I toxins: ApA and ApB from *Anthopleura xanthogrammica* [11,12], APE1–APE5 from *Anthopleura elegantissima* [13]. At the same time, recent investigations of the diversity and functional consequences of type I toxins [14] underscore the substantial difference in the physico-chemical properties and spatial structure of the type I and type II toxins [15].

The structural type II includes ShI from *Stichodactyla helianthus* [9], RpI–RpIV from *Heteractis magnifica* (= *Radianthus paumotensis*) [8], and RTX-I–RTX-V from *Heteractis crispa* (= *Radianthus macrodactylus*) [10,16,17,18]. Electrophysiological studies of ShI and RpI–RpIV [8,19] allowed the determination of a common mechanism of their action on Na_V_, but not a pharmacological profile. No in-depth electrophysiological characterizations of *H. crispa* toxins have been carried out to date.

All sea anemone toxins are suggested to bind within site 3 of Na_V_. Their binding site is assumed to partially overlap with that of scorpion α-toxins and spider δ-toxins, regardless of their different folds. Sea anemone toxins may influence the voltage dependency of inactivation and activation and also slow down the inactivation process, which results in sustained non-inactivating currents.

Peptide association to the ion channels is due to electrostatic interactions between amino acid residues of the toxin molecule and negatively charged residues within an extracellular link connecting the third and fourth transmembrane segments of the Na_V_ domain IV [4].

In the current paper we describe the isolation, structural, and functional characterization of two known type II toxins, δ-SHTX-Hcr1f (= RpII), RTX-III, and a new one, RTX-VI, from the sea anemone *H. crispa*. Electrophysiological screening of these three toxins on Na_V_ channel subtypes showed current activation of the CNS Na_V_ (Na_V_1.1–Na_V_1.3, Na_V_1.6), insect and arachnid (BgNa_V_1, VdNa_V_1) channels. The theoretical model of the sea anemone toxin δ-SHTX-Hcr1f with the rat sodium channel 1.2 (rNa_V_1.2) demonstrates that, despite the overlapping of the sea anemone toxin binding site with that of the scorpion α-toxin, the mode of action on the voltage-sensing domain IV (VSD-IV) is distinct.

## 2. Results

### 2.1. Isolation of the Toxins

**Figure 1:**
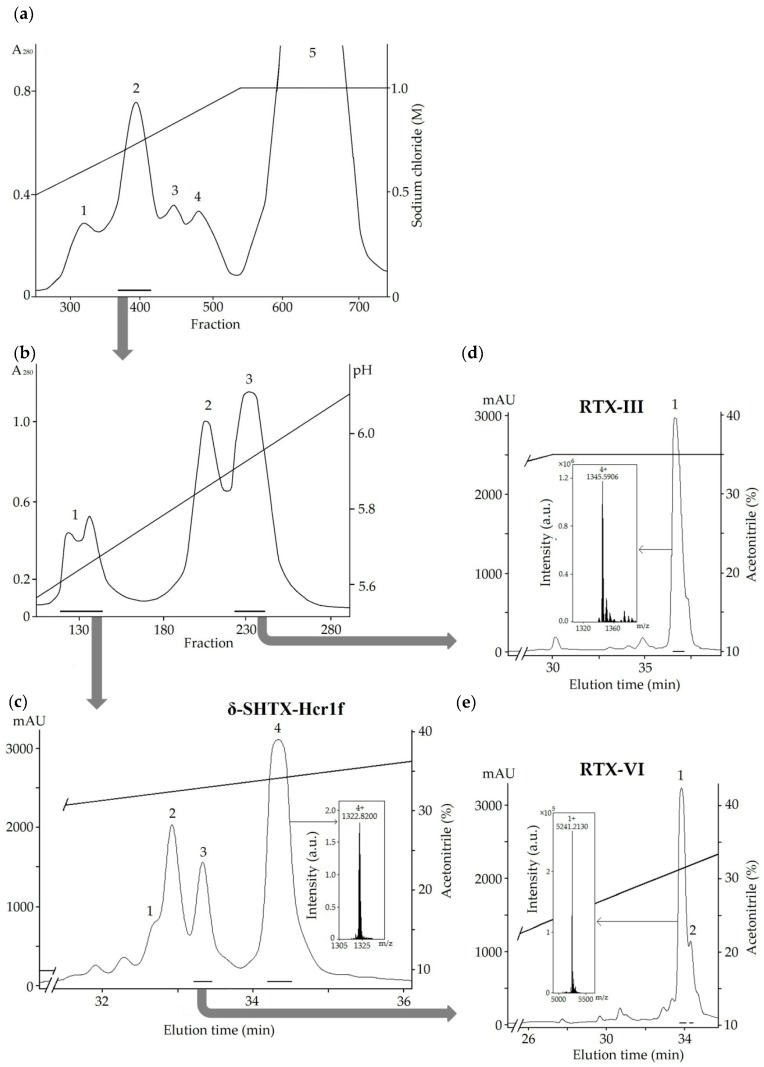
Purification of *Heteractis crispa* toxins. (**a**) Ion-exchange chromatography of the peptide fraction obtained after hydrophobic chromatography [20] was carried out on Bio-Rex 70 column (2.5 × 60 cm); (**b**) Ion-exchange chromatography of fraction 2 was carried out on SP-Sephadex C-25 column (2.5 × 50 cm). (**c**–**e**) RP-HPLC of fractions 1 and 3 on Luna C18 (10 × 250 mm) column. Insets: ESI mass spectra, average molecular masses of peptides are shown. Collected fractions are accentuated by solid lines.

After extraction, peptides were eluted with 20% aqueous ethanol from a Polychrome-1 [20] and separated by cation-exchange chromatography on a Bio-Rex 70 column (Figure 1a), resulting in three distinct fractions. Fraction 2 (Figure 1a), enriched with peptides of molecular masses about 5200 Da according to the mass spectrometry data, was then fractionated on a SP-Sephadex C-25 column (Figure 1b). The separation of fractions 1 and 3 (Figure 1b) by RP-HPLC resulted in peptides with molecular masses corresponding to the type II toxins RpII (δ-SHTX-Rpa1a UniProt ID: P01534; detected molecular mass of 5287.25 Da) [21] and RTX-III (δ-SHTX-Hcr1a, UniProt ID: P30832; 5378.33 Da) [10] (Figure 1c,d) previously derived from the sea anemones *H. magnifica* and *H. crispa*, respectively. The molecular mass of another peptide, RTX-VI (5240.21 Da), isolated by RP-HPLC from fraction 3 (Figure 1c), did not correspond to the mass of any previously described toxin from the *Heteractis* genus (Figure 1e).

### 2.2. Structure Determination

**Figure 2:**
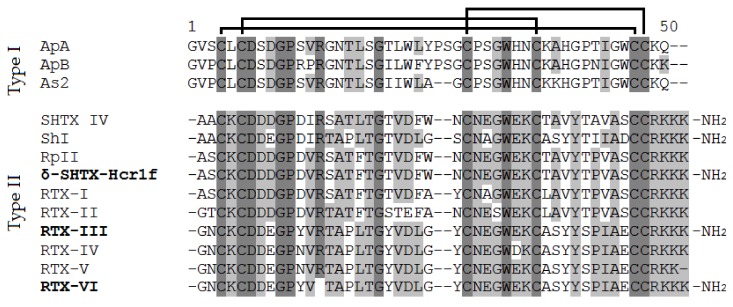
Multiple sequence alignment of type I and II toxins: Ap-A (UniProt ID: P01530) and Ap-B (P01531) from *Anthopleura xanthogrammica*, As2 (P01528) from *Anemonia sulcata*, SHTX IV (B1B5I9) from *Stichodactyla haddoni*, RpII (P01534) from *Heteractis magnifica*, RTX-I (P30831), RTX-II (P30783), RTX-III (P30832), RTX-IV (P30784), RTX-V (P30785), and RTX-VI from *H. crispa,* and ShI (P19651) from *Stichodactyla helianthus*. Identical and conserved amino acid residues are shown on a dark and light gray background, respectively. The amidation of the C-terminal amino acid of neurotoxins is marked based on the literature (Sh1 [22] and SHTX IV [23]) and our results (δ-SHTX-Hcr1f, RTX-III, and RTX-IV). Since amidation of C-termini of Rp-II and RTX-I–RTX-V was not previously declared by Schweitz et al. [8] or Zykova et al. [10,16,17,18] they were considered as unmodified.

To determine the complete amino acid sequences of the three isolated peptides, they were alkylated with 4-vinylpyridine, separated by RP-HPLC, and sequenced by Edman degradation or tandem mass spectrometry. The sequence of the peptide δ-SHTX-Hcr1f isolated from fraction 4 (Figure 1c) was identical to the previously described peptide RpII (Figure 2). RTX-III was identified based on its monoisotopic molecular mass (5378.33 Da) and N-terminal amino acid sequence 1GNCKCDDEGPYV12, which differs from those of the type II homologs.

**Figure 3:**
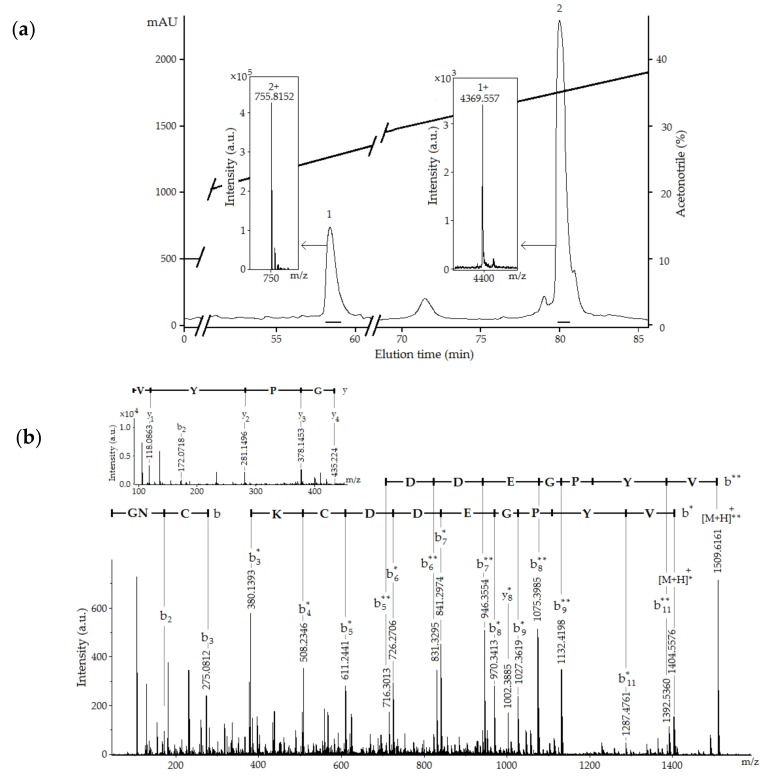
(**a**) RP-HPLC of reduced and alkylated RTX-VI on Nucleosil C18 (4.6 × 250 mm) column illustrating the separation of two peptide chains. Collected fractions are accentuated by solid lines. Insets: ESI mass spectra, average molecular masses of peptides are shown; (**b**) CID spectrum of [M+H]^+^ ion of N-terminal fragment (1–12 aa) of alkylated RTX-VI: GNCKCDDEGPYV. The presence of one or two pyridylethyl groups of Cys marked with * or **, respectively. The y-and b-type ions series are noted above the mass spectrum.

As a result of alkylated RTX-VI separation, we obtained two peaks with average molecular masses of 1509.61 and 4368.55 Da (Figure 3a) instead of one peak of modified peptide with a molecular mass of 5877.09 Da corresponding to the modification of six cysteine residues, as was observed for δ-SHTX-Hcr1f and RTX-III. Sequencing of the native RTX-VI revealed that this peptide is a two-chain molecule: Gly/Thr–Asn/Ala–Xaa/Pro. Further analysis provided unambiguous evidence for the existence of a two-chain peptide. Figure 3b shows the CID spectrum for a single-charged ion [M+H]^+^ of the peptide 1 (1509.61 Da) (Figure 3a). All b-type ions were detected except for b_1_^+^ (Gly1-Asn2) and b_10_^+^ (Pro10-Tyr11). At the same time, y_2_^+^ ion (with the monoisotopic molecular mass of 281.1496 Da), indicating fragmentation between Pro10 and Tyr11, was identified. The following sequence was determined based on the obtained MS/MS data: GNCKCDDEGPYV. This sequence is completely identical with the N-terminal sequence of RTX-III (1–12 aa).

The CID spectra of molecular ions of the polyprotonated peptide 2 (4368.55 Da) were uninformative and yielded no useful structural information. Its amino acid sequence was determined by Edman degradation. The obtained sequence of 35 residues was as follows: TAPLTGYVDLGYCNEGWEKCASYYSPIAECCRKKK. This corresponds to the C-terminal sequence of RTX-III (14–48 aa).

The obtained results indicate a modification of six Cys residues of the RTX-VI molecule consisting of two peptide chains connected by two disulfide bridges. The analysis of the primary structure of both peptides showed that their sequences were completely identical to the corresponding fragments of the RTX-III molecule, with the exception of the Arg13 residue, which is absent in RTX-VI. This observation strongly suggests that RTX-VI is a unique natural modification of the RTX-III toxin. According to the mass-spectrometry data, all *H. crispa* toxins contain an amide on the C-terminus of the molecule, which has not been reported previously. This is a common post-translational modification (removal of the C-terminal Gly and subsequent amidation of the Lys48) occurring during toxin maturation [23].

### 2.3. Secondary Structures of *Heteractis* Toxins

**Figure 4:**
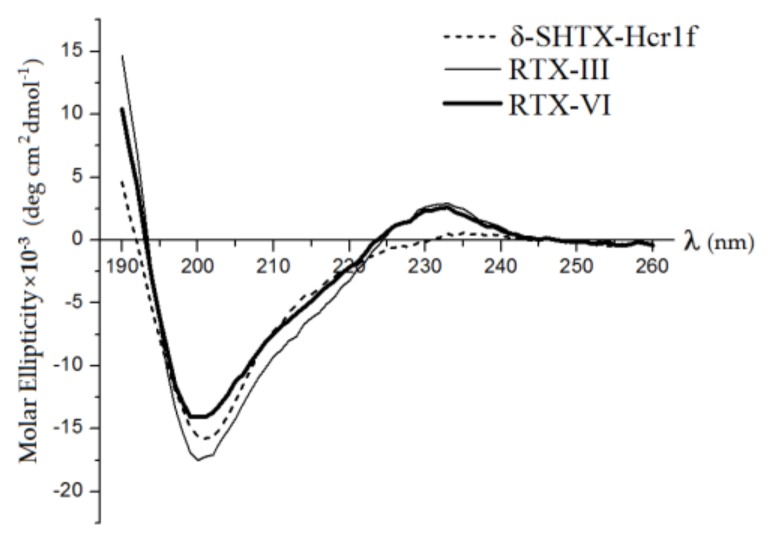
CD spectra of *H. crispa* toxins in double-distilled water at 20 °C. The CD spectra of peptides are shown as dotted (δ-SHTX-Hcr1f), solid (RTX-III), and solid bold (RTX-VI) lines.

We used CD spectroscopy to establish and analyze the secondary structures of isolated toxins. Figure 4 illustrates the CD spectra of the toxins δ-SHTX-Hcr1f, RTX-III, and RTX-VI. Similar to CD spectra of the type I and II toxins [24,25,26,27], those of δ-SHTX-Hcr1f, RTX-III, and RTX-VI had a large negative ellipticity in the vicinity of 200 nm and positive ellipticity around 230 and 190 nm. This indicates that there are no differences in the secondary structure of these toxins.

In general, the CD spectra of δ-SHTX-Hcr1f, RTX-III, and RTX-VI indicated a predominant content of β-strands. According to the NMR data, there are no α-helices in the spatial structure of the previously investigated toxins of types I and II: ApA (PDB ID: 1Ahl) [28], ApB (1Apf) [29], As1 (1Atx) [30], CgNa (2H9x) [31], and ShI (1Shi, 1Sh1, and 2Sh1) [32]. CD spectroscopy of δ-SHTX-Hcr1f, RTX-III, and RTX-VI toxins showed α-helices to be practically absent in their structure (there is 5% of α-helix in δ-SHTX-Hcr1f structure), in accordance with the literature data.

The estimation of the secondary structure content showed that a significant part (from 35% to 40%) of the peptide chains of the toxins adopt a β-conformation (Table 1). Each molecule has an unordered structure ranging from 36% to 40%. Three loops connecting β-strands in the spatial structure of the toxins explain this observation. The rest of the molecule (from 18% to 23%) are β-turns, which are also characteristic elements of the spatial structure of toxins [9].

### 2.4. Electrophysiological Effects on Na_V_ Channels 

Electrophysiological experiments on seven mammalian (Na_V_1.1–Na_V_1.6, Na_V_1.8), one insect (BgNa_V_1), and one arachnid (VdNa_V_1) Na_V_ subtypes were performed in order to determine the pharmacological profiles of δ-SHTX-Hcr1f, RTX-III, and RTX-VI. Toxins were applied at a concentration of 10 μM on Na_V_ channels expressed in *Xenopus laevis* oocytes. Each of three toxins was found to have a unique pharmacological profile, and the observed effects were consistent with the previously described biophysical properties of sea anemone toxins targeting Na_V_ channels [4,9].

**Figure 5:**
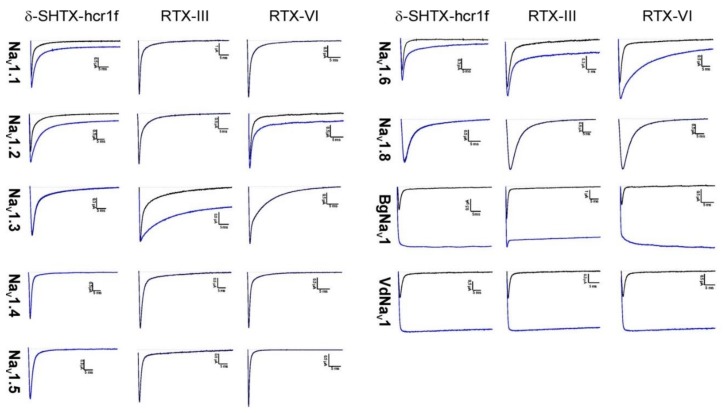
Electrophysiological profile of *Heteractis* toxins on Na_V_ subtypes expressed in *Xenopus laevis* oocytes. Panels show superimposed current traces in control conditions (black traces) and in the presence of 10 μM of peptides (blue traces). The dotted line indicates zero current level. Na^+^ current traces were evoked by 100-ms depolarizations to V_max_ (the voltage corresponding to maximal Na^+^ current in control conditions).

δ-SHTX-Hcr1f, RTX-III, and RTX-VI toxins modulate insect, arachnid (BgNa_V_1, VdNa_V_1), and mammalian CNS Na_V_ channels (Na_V_1.1–Na_V_1.3, Na_V_1.6) but not skeletal muscle (Na_V_1.4), cardiac (Na_V_1.5), or PNS (Na_V_1.8) channels (Figure 5, Table 2). It seems that the absence of Arg13 in the structure of RTX-VI has abolished its effect on the Na_V_1.3 current, since the RTX-III molecule, differing from RTX-VI only by Arg13, exhibited substantial activating effects on Na_V_1.3 that was not seen for RTX-VI. In addition, it was shown that peptide RTX-VI, unlike RTX-III, modulated another subtype of the CNS channels, Na_V_1.2.

**Figure 6:**
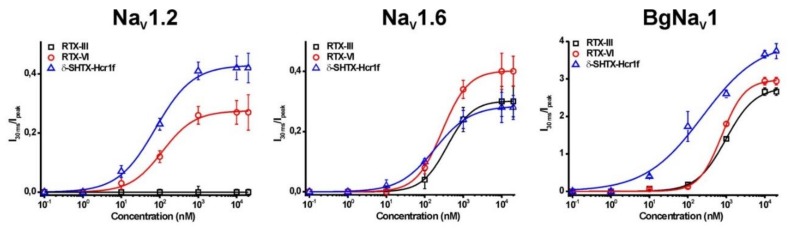
Concentration–response curves for Na_V_1.2, Na_V_1.6, and BgNa_V_1, indicating the concentration dependence of δ-SHTX-Hcr1f (blue), RTX-III (black), and RTX-VI (red) induced effects. The same protocols as described in Figure 5 were used to construct the concentration–response curves.

**Figure 7:**
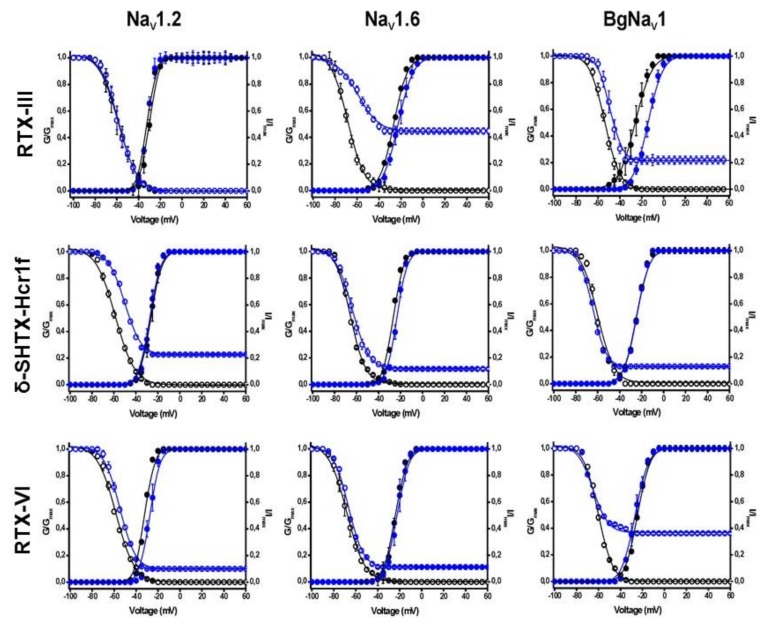
Steady-state activation (closed symbols) and inactivation (open symbols) curves in control condition (black) and in the presence of 100 nM of toxin (blue). The current–voltage relationships were determined by 50-ms step depolarizations between −90 and 70 mV, using 5 mV increments. Toxin-induced effects on the steady-state inactivation were investigated by using a standard two-step protocol. In this protocol, 100 ms conditioning 5 mV step prepulses ranging from −90 to 70 mV were followed by a 50 ms test pulse to −30 or −10 mV.

Similar to many other sea anemone toxins [33,34,35,36], δ-SHTX-Hcr1f, RTX-III, and RTX-VI appeared not to be phyla-selective (mammalian vs. insect/arachnid). When both mammalian and insect or arachnid subtypes were treated by the same concentration of toxins (10 μM) there was little if any inactivation of insect or arachnid channels to be observed (Figure 5). At the same time, the EC_50_ value for mammalian channels was substantially lower than those for insect or arachnid channels (Figure 6, Table 3). The increase of the current amplitude (both fast and slow components) is associated with an incomplete inactivation process and positive shift of the voltage dependence of the inactivation process (Figure 7).

### 2.5. Toxicity of *Heteractis* Toxins

Testing of toxicity in mice showed that the activity of δ-SHTX-Hcr1f was consistent with the previously reported data for RpII (LD_50_ 4200 μg/kg) [8]. RTX-III showed a very high toxicity (LD_50_ 2.5 μg/kg) [10], but activity disappeared in the case of RTX-VI (LD_50_ > 10 mg/kg). The only difference observed within our electrophysiological experiments that may underlie this phenomenon is a different activity of both toxins on Na_V_1.2 and Na_V_1.3 subtypes (Figure 5, Table 2). Even though tetrodotoxin (TTX)-sensitive brain-type sodium channel isoforms Na_V_1.1–Na_V_1.3 and Na_V_1.6 are the main channels expressed in the CNS, their participation in other physiological processes is still under investigation. Recent data provide evidence that Na_V_1.1, Na_V_1.3, and Na_V_1.6 play an important role in action potential propagation in the cardiomyocyte and in excitation–contraction coupling [37,38]. However, further research is needed to verify if the strong toxic effect of the RTX-III toxin comes solely from the modulation of Na_V_1.3 channels.

### 2.6. Homology Models of *Heteractis* Toxins

The models of the 3D structure of δ-SHTX-Hcr1f (= RpII, UniProt ID: P01534) and RTX-III (UniProt P30832) were created, using as template a spatial NMR structure of the closest homolog of these toxins, type II neurotoxin from the sea anemone *S. helianthus*, ShI (PDB ID 2Sh1) [32] (sequences share 67% identity). The obtained theoretical models had no conformational hindrances, and the content of the secondary structure elements was in acceptable agreement with the CD spectroscopy data (Table 1), indicating the high accuracy of the generated models. 

To consider the possibility of disulfide bond pattern changing in RTX-VI, the homology models of RTX-III and a set of RTX-VI 3D-models with various disulfide bond topologies were also generated (data not shown). The only RTX-VI theoretical model that adopted a β-defensin fold typical for type I and II toxins (with C3-C43, C5-C33, and C26-C44 bonds) was found to be in perfect agreement with the CD spectroscopy data for both toxins (Table 1).

### 2.7. Molecular Modeling of δ-SHTX-Hcr1f Interaction with rNa_V_1.2

Homology models of rNa_V_1.2 (UniProt ID: P04775) [39] including extracellular loops, pore-forming, and voltage-sensing domains were generated. The 3D structure of the human sodium channel Na_V_1.4 (PDB ID: 6AGF), determined by cryo-electron microscopy, was used as a template [40] (sequences share 72% identity). To clarify the possible action mechanism of the toxins on Na_V_, a combination of various protein–protein docking methods was applied to compute the model of δ-SHTX-Hcr1f complex with the rNa_V_1.2.

**Figure 8:**
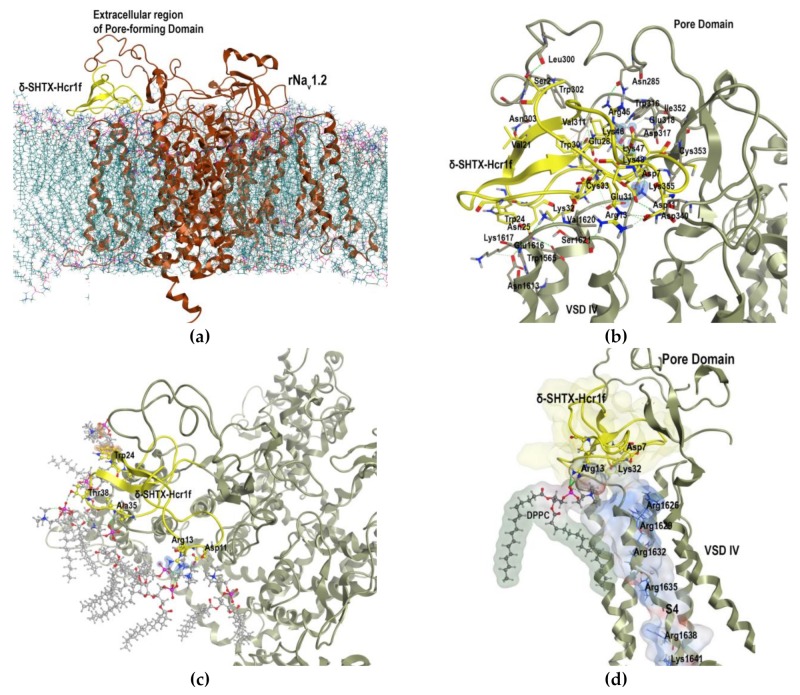
δ-SHTX-Hcr1f toxin interaction with rNa_V_1.2. (**a**) Ribbon diagram of the δ-SHTX-Hcr1f–rNa_V_1.2 complex (ribbons for both) embedded into dipalmitoylphosphatidylcholine (DPPC) bilayer (sticks); (**b**) Direct intermolecular interactions in the complex: side chains of toxin and rNa_V_1.2 involved in binding (sticks); (**c**) δ-SHTX-Hcr1f interacts with phospholipids (bold and sticks for both) at the region of the pore and the VSD-IV (top view); (**d**) Arg13 contacts with phospholipid adjacent to the S4 helix of rNa_V_1.2. The Arg13 side chain and the phospholipid atoms are presented as balls and sticks, the molecular surfaces of δ-SHTX-Hcr1f and a phospholipid are presented as solid surfaces, colored light orange and according to lipophilicity, respectively, whereas the molecular surface of the S4 helix is presented as a solid surface colored according to electrostatic property (front view). Part of the rNa_V_1.2 structure has been removed for clarity. δ-SHTX-Hcr1f (yellow), rNa_V_1.2 (gray green), and DPPC bilayer (gray), hydrogen bonds (green dotted line); ionic and cation-π interactions are represented as blue and orange colored contours, respectively.

According to the analysis of these models, the δ-SHTX-Hcr1f binding site is located mainly at the area of the extracellular region of the rNa_V_1.2 VSD-IV (Figure 8). It includes Glu1616, Lys1617, and Val1620 of the sodium channel that form interactions with the charged residues Asp6, Arg13, and Lys32 of δ-SHTX-Hcr1f. Furthermore, residues of the extracellular region of the pore-forming domain I, Asp317, Lys355, Gly318, Asn340, and Asn285, interact with residues Lys46, Lys48, Glu31, Asp7, and Arg45 of the toxin.

To extend our understanding of the interaction driving forces and a possible mode of the toxin action on the channel, full atomic molecular dynamics (MD) simulations of the δ-SHTX-Hcr1f–rNa_V_1.2 complex embedded into lipid bilayer in an aqueous environment were carried out (Figure 8a). The results of MD simulations revealed significant conformation changes in the channel structure during interaction with the toxin. Thus, the root-mean-square deviation (RMSD) value of Cα atoms of the pore-forming and the VSD-IV domains were 4.5 Å, with the greatest changes in the channel conformation affecting the pore domain extracellular region with the maximum deviation observed for Glu304 and Thr305 residues whose RMSD of Cα atoms reached 20.52 Å and 18.01 Å, respectively. It should be noted that such changes in the channel conformation were not detected in the case of the RTX-III toxin.

According to the calculated data, the charged C-terminal residues (Lys48, Lys46, Glu31) and Asp7 coordinate the toxin’s orientation at the extracellular region of the rNa_V_1.2 pore domain through a network of electrostatic interactions and hydrogen bonds. These residues are assumed to be mainly responsible for the binding of δ-SHTX-Hcr1f to this channel subtype, since they make a maximum contribution of −71.39, −42.289, −26.446, and −23.855 kcal/mol, respectively (Table S1). At the same time, the other group of polar residues such as Lys32, Asn25, Arg13, and hydrophobic Cys33 interact with VSD-IV with much lower affinity compared to the former group, and their contribution ranges from −14.48 kcal/mol, for Lys32 binding both Glu1616 at the S3 and Ser1621 at the S4 helices of VSD-IV, to −1.164 kcal/mol for Cys33 that forms two hydrogen bonds with Val1620 and Ser1621 at the base of the S4 helix VSD-IV simultaneously (Table S1).

MD simulations also revealed a set of δ-SHTX-Hcr1f contacts with phospholipids adjacent to the channel at the region of the pore and the VSD-IV (Figure 8c,d). The Arg13 side chain of the toxin binds quite strongly the surrounding VSD-IV phospholipid’s head by two hydrogen bonds and three salt bridges with a total contribution of −18.80 kcal/mol in addition to hydrophobic interactions (Figure 8d, Table S2). Four other residues, Asp11, Val36, Thr38, and Trp24, contribute less to the stabilization of the complex: −5.35, −7.22, −2.99, and −2.91 kcal/mol, respectively (Figure 8c).

## 3. Discussion

The type I and II toxins are one of the most-studied groups of sea anemone peptides as they constitute a majority of the venom components [9,41]. Despite this fact, few toxins have been studied in terms of their ability to distinguish closely related Na_V_ subtypes. In this work we conducted an in-depth investigation of two known type II *Heteractis* toxins—RTX-III [10] and δ-SHTX-Hcr1f (=RpII [21])—as well as a new one, RTX-VI. All of them were isolated from 20% aqueous-ethanolic fraction by hydrophobic chromatography on a Polychrome-1 [20] and purified using two-step cation exchange chromatography followed by RP-HPLC.

The unique natural analogue of RTX-III, the toxin RTX-VI, consists of two peptide chains (12 and 35 aa) connected by two disulfide bonds (C3–C43 and C5–C33); the third disulfide bond is formed within chain 2 (C26–C44). Deletion of Arg13 in RTX-VI does not lead to significant changes in its secondary structure, characteristic for the type I and II toxins [31]. RTX-VI appears to be the result of a previously undescribed post-translational modification that occurs in the sea anemone rather than during isolation of the peptide. The processing quadruplet motif (PQM) protease participating in the maturation of two-chain spider toxins and performing cleavage of the C-termini of Arg was identified [42]. However, there is no processing site of this protease in the sequence of RTX-III. Therefore, the mechanism of the observed modification and its biological relevance are unknown.

The role of the highly conserved residue Arg13/14 (numbering for type II/I toxins, respectively) localized at the top of the arginine loop is a discussion point of functional investigations of sea anemone type I and II toxins. The arginine loop was shown to be crucial for the affinity and selectivity towards Na_V_ channels [9,26].

According to the obtained electrophysiological data, δ-SHTX-Hcr1f, RTX-III, and RTX-VI toxins did not affect currents of skeletal muscle Na_V_1.4, cardiac Na_V_1.5, and PNS Na_V_1.8 channels (Figure 5, Table 2). They did slow down the inactivation of CNS channels of mammalian (Na_V_1.1–Na_V_1.3, Na_V_1.6), insect (BgNa_V_1), and arachnid (VdNa_V_1) channels. RTX-VI, devoid of Arg13, residue differently, in comparison with RTX-III, affected the Na_V_1.2 and Na_V_1.3 current (Figure 5, Table 2). While RTX-VI enhanced current through Na_V_1.2, RTX-III acted similarly on Na_V_1.3. Taking into account the effects of RTX-III and RTX-VI on Na_V_ inactivation, we believe that Arg13 contributes to the discrimination between Na_V_ isoforms rather than being crucial for the toxin–channel interaction.

At the same time, RTX-VI is a more potent inhibitor of both Na_V_1.6 and BgNa_V_1 channels than RTX-III, which suggests that removal of the Arg13 or peptide chain cleavage may favor toxin–channel interaction. Khera and co-authors [43] emphasized that the presence of at least one positively charged residue in the arginine loop is necessary for a toxin binding with a channel and for subsequent stabilization of Na_V_ open state. Arg14 was found to be significantly less important than Arg12 in ApB from *A. xanthogrammica*. The substitution of Arg14Gln in ApB can be compensated by Arg12 and a positive charge at the C-terminus of the molecule. In general, δ-SHTX-Hcr1f, RTX-III, and RTX-VI toxins are non-phyla-selective. Nonetheless, they effectively potentiate insect and arachnid channels with high EC_50_ values and potentiate mammalian channels less effectively but with low EC_50_ values. δ-SHTX-Hcr1f is the most potent inhibitor of Na_V_ channels among three toxins (δ-SHTX-Hcr1f > RTX-VI > RTX-III), and their preferential targets are Na_V_1.2 > Na_V_1.6 > BgNa_V_1.

Our results derived from a molecular modeling approach showed that the sea anemone toxin δ-SHTX-Hcr1f forms a network of strong interactions of predominantly charged residues with the binding site at the VSD-IV region overlapping with that of the scorpion α-toxin AaH2 [44]. MD simulations of the δ-SHTX-Hcr1f complex with rNa_V_1.2 revealed a distinction in the mode of their action. Cryo-EM experiments showed that the Arg62 residue of AaH2 enters deep into the pore between VSD helices and reaches 11 Å from the R1 gating charge of chimeric VSD-IV-Na_V_Pas channel, preventing the S4 helix from moving “up” and trapping it in the inactivated state [44]. In contrast, Arg13 and Lys32 of the sea anemone toxin δ-SHTX-Hcr1f do not penetrate deep into the pore between S1 and S4 helices but are located at a distance of more than 14.5 Å from the channel R1 and R2 gating charges. The side chain of Lys32 is involved in a whole set of strong hydrogen bonds with the S3–S4 helix linker, namely with Glu1616 (−8.57 kcal/mol) and Ser1621 (−5.91 kcal/mol) (Figure 8b,d). Arg13 forms direct contact with the VSD-IV domain, namely a hydrogen bond (−2.127 kcal/mol) with Val1620 localized at the beginning of the channel S4 helix (Figure 8b). Moreover, Arg13 is able to interact with a phospholipid which is very closely adjacent and is strongly associated with both the pore domain I and the S4 helix of the channel (Figure 8c,d, Table S2). An additional toxin interaction with the channel phospholipid environment contributes to the fixation at the channel binding site. It should be noted that the RTX-VI toxin without the Arg13 residue exhibited a lower activity on the Na_V_1.2 subtype compared to δ-SHTX-Hcr1f (Figure 6, Table 3).

An analysis of the channel conformational changes revealed that the binding of δ-SHTX-Hcr1f with the rNa_V_1.2 channel causes significant conformational changes of the extracellular region of the pore domain I and less-significant changes in VSD-IV. At the S1–S4 region, the R gating charge motif moves 1.82–3.84 Å downward and toward the channel pore. However, its position relative to the hydrophobic constriction site remains characteristic for a channel in the activated state [45]. It seems that the S3–S4 loop, being bound by the δ-SHTX-Hcr1f toxin, partially loses its inherent mobility, and this prevents free motion of the S4 helix relative to other helices in the VSD-IV domain for the channel transition from the activated to the inactivated state. Taken together, the results obtained from the molecular modeling are in good agreement with the functional data of δ-SHTX-Hcr1f obtained in the electrophysiological experiments.

## 4. Materials and Methods 

The specimens of *H. crispa* were collected from the South China Sea, Vietnam (2013). The species of sea anemone were identified by Dr. E. Kostina (A.V. Zhirmunsky National Scientific Center of Marine Biology FEB RAS). Sea anemones were frozen and kept at −20 °C.

### 4.1. Extraction and Chromatographic Procedure

Peptides were extracted from the sea anemone *H. crispa* using 70% ethanol and separated by hydrophobic chromatography on a Polychrome-1 (powdered Teflon, Biolar, Olaine, Latvia) column (4.8 × 95 cm) using a step gradient of ethanol concentration at a flow rate of 120 mL/h and fraction size of 20 mL [20]. The hydrophobic 20% aqueous-ethanolic fraction was further separated by cation-exchange chromatography on Bio-Rex 70 column (2.5 × 60 cm) (Bio-Rad, Richmodn, CA, USA) equilibrated with an ammonium acetate buffer (pH 4.5) at a flow rate of 22 mL/60 min and a fraction size of 5.8 mL, followed by cation-exchange chromatography on Sephadex C-25 (2.5 × 40 cm) (Amersham Biosciences, Piscataway, NJ, USA) at a flow rate of 70 mL/60 min and a fraction size of 7 mL. A linear gradient of NaCl concentration (solved in a 5 mM ammonium acetate buffer) from 0 to 1 M and those of pH from 5.5 to 6.5 (a 100 mM ammonium acetate buffer) were used, respectively. The protein concentration was determined by Lowry method, bovine serum albumin was used as a standard [46]. 

Next, neurotoxic fractions were separated on a reversed-phase Luna C18 column (10 × 250 mm) equilibrated with 10% acetonitrile solution in 0.1% trifluoroacetic acid (TFA) on an Agilent 1100 chromatograph (Agilent Technologies, Santa Clara, CA, USA). Peptide elution was carried out using a linear gradient of acetonitrile concentration (with 0.1% TFA and at a flow rate of 3 mL/min) according to the scheme: 10–40% of acetonitrile for 40 min. The final separation of the active peptides obtained after RP-HPLC was made on the same column in two alternative gradients of acetonitrile concentration (with 0.1% TFA and at a flow rate of 3 mL/min): 10% of acetonitrile for 5 min, 10–35% of one for 25 min, then 35% for 10 min or 10% of one for 5 min, 10–35% for 35 min. The Vacuum concentrator 5301 (Eppendorf, Germany) was used for acetonitrile evaporation. 

### 4.2. Mass Spectrometric Analysis

A mass spectrometric analysis was carried out using an Ultraflex TOF/TOF mass spectrometer (Bruker Daltonik, Germany). The samples were solved in acetonitrile/water solution (1:1, *v/v*) containing 0.1% TFA and mixed with 10 mg/mL of sinapinic acid as a matrix. Protein molecular masses (5 × 10^3^–2 × 10^4^ Da) were obtained in linear or reflector mode with external calibration. 

### 4.3. Tandem Mass Spectrometry (MS/MS)

The amino acid sequence of peptide fragments of RTX-VI (1–12 aa) treated with 4-vinylpyridine was identified from collision-induced dissociation (CID) tandem mass spectra. CID MS/MS experiments were performed on a MaXis impact ultra-high-resolution quadrupole time-of-flight mass spectrometer (Bruker Daltonik, Germany) equipped with an ESI ionization source. A survey mass spectrum and a tandem mass spectrum were recorded for each sample. During MS/MS, the fragment ions were generated from the isolated [M+H]^+^ peptide precursor ion by low-energy CID with collision energies of 25, 35 and 95 eV.

### 4.4. Reduction and Alkylation of Disulfide Bridges

Peptides were reduced and alkylated with 4-vinylpyridine, as described in [46]. Separation of the reaction mixture was made on a reversed-phase Nucleosil C18 column (4.6 × 250 mm) equilibrated with 10% acetonitrile in 0.1% TFA. The elution was carried out using a combined gradient of acetonitrile concentration (with 0.1% TFA and at a flow rate of 0.5 mL/min): 10% acetonitrile for 30 min and then 10–40% for 60 min.

### 4.5. Sequence Determination

Peptides were reduced with dithiothreitol and alkylated with 4-vinylpyridine prior to N-terminal automated Edman sequencing, using Procise 492 cLC protein sequencing system (Applied Biosystems, Foster City, CA, USA). The sequence homology was analyzed using amino acid sequence databases and the BLAST (Basic Local Alignment Search Tool) algorithm [47]. Multiple alignment of amino acid sequences was made using Vector NTI software (Invitrogen, Carlsbad, CA, USA) [48].

### 4.6. Circular Dichroism Spectra

CD spectra were measured in the range from 320 to 190 nm with a Chirascan-plus spectropolarimeter (Applied Photophysics, Leatherhead, UK). The instrument was calibrated with 10-camphorsulfonic acid ammonium salt, and a ratio of 2:1 was found between the positive CD band at 290 nm and the negative CD band at 192 nm. The measurements were carried out in bidistilled water at 20 °C using 0.1- and 1-cm path length cells in amide and aromatic regions, respectively. In the amide region (190–240 nm) of the CD spectrum, molar ellipticity [θ]_λ_ (deg cm^2^ dmol^−1^) was calculated as [θ]_λ_ = θ_obs_ × MRW/10 × C × L, where θ_obs_ is the observed ellipticity in degrees, MRW is the mean amino acid residue weight of 110 Da, L is the path length (cm), and C is the protein concentration (g/mL). In the aromatic region (240–320 nm) of the CD spectrum, results were expressed as molar ellipticity (the average molecular masses of RpII, RTX-III, and RTX-VI are 5378, 5287, and 5240 Da, respectively). The secondary structure contents were determined according to the method of Provencher and Glöckner, using their CONTIN program [49].

### 4.7. Toxicity

The care and use of animals were performed in an agreement with the requirements of local and international legislation as well as of the Directive 2010/63/EU for animal experiments. 

Adult male CD-1 mice weighing 20–22 g were kept at room temperature with a 12-h light/dark cycle and with ad libitum access to food and water. Each animal was used only once and was euthanized in the CO_2_ chamber immediately after completion of the experiment. Animal procedures were approved by the Animal Care and Use Committee of the PIBOC FEB RAS.

The peptides (0.15–300 μg) were dissolved in 100 μL of sterile saline (0.9% NaCl) and injected intraperitoneally. Six animals were used per sample. To assess peptide toxicity, the maximum non-toxic dose was determined [50] as an amount (mg) of the tested peptide per kg of the mouse weight, at which there was no observed toxic or adverse effect within 24 hours.

### 4.8. Expression of Voltage-Gated Ion Channels in *Xenopus laevis* Oocytes

For the expression of Na_V_ channels (hNa_V_1.1, rNa_V_1.2, rNa_V_1.3, rNa_V_1.4, hNa_V_1.5, mNa_V_1.6, rNa_V_1.8, the invertebrate channels BgNa_V_1.1, VdNa_V_1, and the auxiliary subunits rβ1, hβ1, and TipE) in *Xenopus* oocytes, the linearized plasmids were transcribed using the T7 or SP6 mMessage-mMachine Transcription Kit (Thermo Fisher Scientific, Waltham, MA, USA). The harvesting of stage V–VI oocytes from anesthetized female *X. laevis* frogs was previously described [51] with a protocol adjustment in which the frogs were anesthetized by placement in 0.1% tricaine (amino benzoic acid ethyl ester; Millipore Sigma, Burlington, MA, USA) solution. Oocytes were injected with 50 nL of cRNA at a concentration of 1 ng/nL using a microinjector (Drummond Scientific Company, Broomall, PA, USA). The oocytes were incubated in a solution containing (mM) the following: NaCl, 96; KCl, 2; CaCl_2_, 1.8; MgCl_2_, 2; and HEPES, 5 (pH 7.4). This solution was supplemented with 50 mg/L gentamicin sulfate.

### 4.9. Electrophysiological Recordings

Two-electrode voltage-clamp recordings were performed at room temperature (18–22 °C) using a GeneClamp 500 amplifier (Molecular Devices, San Jose, CA, USA) controlled by a pClamp data acquisition system (Axon Instruments, Union City, CA, USA). Whole-cell currents from oocytes were recorded 1–4 d after mRNA injection. Bath solution composition was (mM) as follows: NaCl, 96; KCl, 2; CaCl_2_, 1.8; MgCl_2_, 2; and HEPES, 5 (pH 7.4). Voltage and current electrodes were filled with 3 M KCl. The resistances of both electrodes were kept between 0.8 and 1.5 MΩ. The elicited currents were filtered at 1 kHz and sampled at 20 kHz by using a 4-pole low-pass Bessel filter. Leak subtraction was performed using a -P/4 protocol. 

For the electrophysiological analysis of toxins, a number of protocols were applied from a holding potential of −90 mV with a start-to-start frequency of 0.2 Hz. Na^+^ current traces were evoked by 100-ms depolarizations to V_max_ (the voltage corresponding to maximal Na^+^ current in control conditions). The current–voltage relationships were determined by 50-ms step depolarizations between −90 and 70 mV, using 5 mV increments. The Na^+^ conductance (g_Na_) was calculated from the currents according to Ohm’s law: g_Na_ = I_Na_/(V − V_rev_), where I_Na_ represents the Na^+^ current peak amplitude at a given test potential V, and V_rev_ is the reversal potential. The values of g_Na_ were plotted as a function of voltage and fitted using the Boltzmann function: g_Na_/g_max_ = [1 + (exp(Vg − V)/k)] −1, where g_max_ represents maximal g_Na_, V_g_ is the voltage corresponding to half-maximal conductance, and k is the slope factor. Toxin–induced effects on the steady–state inactivation were investigated by using a standard two-step protocol. In this protocol, 100 ms conditioning 5 mV step prepulses ranging from −90 to 70 mV were followed by a 50-ms test pulse to −30 or −10 mV. Data were normalized to the maximal Na^+^ current amplitude, plotted against prepulse potential, and fitted by using the Boltzmann function: I_Na_/I_max_ = {(1 − C)/(1 + exp[(V − V_h_)/k)]} + C, where I_max_ is the maximal I_Na_, V_h_ is the voltage corresponding to half-maximal inactivation, V is the test voltage, k is the slope factor, and C is a constant representing a non-inactivating persistent fraction (close to 0 in control). To assess the concentration–response relationships, data were fitted with the Hill equation: y = 100/{1 + [EC_50_/(toxin)]^h^}, where y is the amplitude of the toxin–induced effect at 30 ms (I_30ms_), EC_50_ is the toxin concentration at half–maximal efficacy, (toxin) is the toxin concentration, and h is the Hill coefficient. The degree of inactivation was assayed by measuring the I_30 ms_/I_peak_ ratio, which gives an estimate of the probability for the channels not to be inactivated after 30 ms. Depending on the sodium channel, a test voltage was chosen so that I_30ms_/I_peak_ was close to zero under control conditions. I_30ms_/I_peak_ was measured at the same test voltage after addition of the toxin. The EC_50_ values obtained are summarized in Table 3. All data are presented as means ± sem of at least five independent experiments (n ≥ 5). All data were tested for normality using a D’Agostino–Pearson omnibus normality test. All data were tested for variance using the Bonferroni test or Dunn’s test. Data following a Gaussian distribution were analyzed for significance using one-way ANOVA. Nonparametric data were analyzed for significance using the Kruskal–Wallis test. Differences were considered significant if the probability that their difference stemmed from chance was < 5% (*p* < 0.05). All data were analyzed using pClamp Clampfit 10.0 (Molecular Devices) and Origin 7.5 software (OriginLab, Northampton, MA, USA).

### 4.10. Molecular Modelling

The models of the 3D structure of δ-SHTX-Hcr1f (= RpII, UniProt ID: P01534) [21] and RTX-III were created using UCSF Chimera 1.11.2 [52] with Modeller 9.19 plug-in [53], with the type II neurotoxin from the sea anemone *S. helianthus*, Sh1 (PDB ID 2Sh1) [32] as a template (67% sequences identity). The obtained models were optimized with MOE 2019.0101 CCG^®^ software using Amber12:EHT force field. The restraints on the distance between sulfur atoms of some Cys pairs (2 Å) and templates (exclusion of the type I and II toxins’ spatial structures, PDB ID: 1sh1, 2sh1, 1shi, 2h9x, 1apf, 1ahl, 1atx) for modeling of alternative disulfide-bond isoforms of the toxin RTX-III were imposed. 

Homology models of rNa_V_1.2 (UniProt ID: P04775) [39] were generated with the web-server Swiss-Model [54]. The 3D structure of the human sodium channel hNa_V_1.4–β1 complex (PDB ID: 6AGF, sequences share 72% identity), determined by cryo-electron microscopy at 3.2Å resolution, was used as template [40]. According to the Ramachandran plot analysis, about 92% of the residues of rNa_V_1.2 models generated were found to be in favorable conformations, and 7.6% were in acceptable conformations. This indicates the resulting models can be used for further computational investigation.

A combination of several protein–protein docking techniques allowed us to compute about 4 × 10^4^ (for each pare) possible configurations of the neurotoxin δ-SHTX-Hcr1f complex with rNa_V_1.2 channel. Initially, the web-server Clus Pro 2.0 was used for rigid protein–protein docking [55]. Then ToxDock [56] as well as MOE protein–protein docking algorithms were applied to consider the conformational flexibility of ligand and receptor molecules [57]. All computed docking poses were clustered and analyzed. The most energetically favorable complexes of highly populated clusters that satisfied the modern concept of the sea anemone toxins with Na_V_ channels underwent energy minimization in an Amber12:EHT force field [58] analyzed in greater detail using MOE 19.001 CCG^®^ software [57]. Analysis of the contact surfaces of theoretical complexes and evaluation of binding energy contribution of various amino acid residues were performed with MOE 19.001 CCG^®^ software [57]. The starting states of the δ-SHTX-Hcr1f and RTX-III complexes with rNa_V_1.2 embedded into a DPPC lipid bilayer were constructed with MemProtMD resources [59]. Then, the starting system states were hydrated and neutralized in an Amber12:EHT force field [58], and full-atom MD simulations were performed under conditions of constant pressure, 300 K, and pH 7.0 using MOE 19.1001 CCG® software [57] for 600 ns. Prior to MD simulations, the whole system was heated from 0 to 300 K (200 ps) and equilibrated to reduce initial bad contacts (200 ps). 

## Figures and Tables

**Table 1:** Secondary structure content of *H. crispa* toxins. Estimations of the secondary structure content (percentage) of peptides δ-SHTX-Hcr1f, RTX-III, and RTX-VI. The indices R, D, and tot refer to regular or distorted structures and their sum, respectively.

Peptide	α-Helices			β-Turns			β-Turns	Unordered
	α_R_	α_D_	α_tot_	β_R_	β_D_	β_tot_		
δ-SHTX-Hcr1f	0.4	4.7	5.1	22.6	12.1	34.7	23.5	36.7
RTX-III	0.1	0.4	0.5	27.8	13.3	41.1	18.3	40.1
RTX-VI	0.0	0.3	0.3	26.9	13.6	40.5	20.9	38.3

**Table 2:** Selectivity screening of δ-SHTX-Hcr1f, RTX-III, and RTX-VI on a panel of Na_V_ channels.

Toxin	Voltage-Gated Sodium Channel Subtype								
	Na_V_1.1	Na_V_1.2	Na_V_1.3	Na_V_1.4	Na_V_1.5	Na_V_1.6	Na_V_1.8	BgNa_V_1	VdNa_V_1
δ-SHTX-Hcr1f	+	+	-	-	-	+	-	+	+
RTX-III	-	-	+	-	-	+	-	+	+
RTX-VI	-	+	-	-	-	+	-	+	+

**Table 3:** The half-maximal effective concentration (EC_50_) of δ-SHTX-Hcr1f, RTX-III, and RTX-VI toxins acting on various subtypes of Na_V_ channels.

Toxin	EC_50_ (nM)		
	Na_V_1.2	Na_V_1.6	BgNa_V_1
δ-SHTX-Hcr1f	79.5 ± 4.3	183.5 ± 8.2	226.1 ± 12.3
RTX-III	Not active	381.8 ± 5.8	978.1 ± 62.3
RTX-VI	120.9 ± 19.3	282.3 ± 7.0	760.5 ± 25.6

## Supplementary Materials

The following are available online at https://www.mdpi.com/2072-6651/12/1/44/s1. Table S1: Hot spots of δ-SHTX-Hcr1f interchain non-covalent interactions with NaV1.2; Table S2: δ-SHTX-Hcr1f residue Arg13 non-covalent interchain with phospholipid, adjacent to VSD-IV helix S4.
