# Supplementary material for: New Insights into the Type II Toxins from the Sea Anemone Heteractis crispa
Source: Toxins (Basel). 2020 Jan 10;12(1):44. doi: 10.3390/toxins12010044 (PMC7020476; doi:10.3390/toxins12010044)
Supplement: Supplementary file 1 [file toxins-12-00044-s001.pdf]

Supplementary Materials: New Insights into the Type II Toxins from the Sea Anemone *Heteractis crispa*

Rimma S. Kalina, Steve Peigneur, Elena A. Zelepuga, Pavel S. Dmitrenok, Aleksandra N. Kvetkina, Natalia Y. Kim, Elena V. Leychenko, Jan Tytgat, Emma P. Kozlovskaya, Margarita M. Monastyrnaya and Irina N. Gladkikh

Table S1. Hot spots of δ-SHTX-Hcr1f interchain non-covalent interactions with rNav1.2.

Type[1]	δ-SHTX-Hcr1f	rNav1.2	Energy contribution, kcal/mol	rNav1.2 region
DIH	Lys46	Asp317	-28.909	Eα1a-Eα1b linker
DIH	Lys48	Lys355	-27.764	Eβ1b
DIH	Glu31	Lys355	-26.446	Eβ1b
DIH	Asp7	Lys355	-23.855	Eβ1b
DIH	Lys48	Asp317	-23.828	Eα1a-Eα1b linker
DIH	Lys48	Glu318	-19.798	Eα1b
DIH	Lys46	Glu318	-13.38	Eα1b
DH	Lys32	Glu1616	-8.574	S3 VSD-IV
DH	Lys47	Cys353	-8.414	Eβ1b
DH	Asp7	Asn340	-6.453	Eα1b-Eβ1b linker
DH	Lys32	Ser1621	-5.907	S4 VSD-IV
DH	Arg45	Asn285	-5.338	Eα1a-Eα1b linker
DH	Asn25	Glu1616	-4.618	S3 VSD-IV
DH	Val21	Asn303	-4.282	Eα1a-Eα1b linker
DH	Asn25	Glu1613	-3.713	S3 VSD-IV
DH	Lys46	Trp316	-2.845	Eα1a-Eα1b linker
D	Asp8	Ile352	-2.838	Eβ1b
D	Arg45	Trp316	-2.784	Eα1a-Eα1b linker
DH	Asp11	Asn340	-2.194	Eα1b-Eβ1b linker
DH	Glu28	Val311	-2.139	Eα1a-Eα1b linker
DH	Arg13	Val1620	-2.127	S3-S4 linker, VSD-IV
DH	Ser2	Leu300	-1.981	Eα1a
D	Trp30	Trp302	-1.515	Eα1a
D	Val36	Lys1617	-1.492	S3 VSD-IV
D	Trp24	Trp1565	-1.47	S2 VSD-IV
D	Asp6	Val1620	-1.238	S3-S4 linker, VSD-IV
DH	Asp8	Cys353	-1.199	Eβ1b

[1]H - hydrogen bonds, I - ionic, D - distance (hydrophobic, van der Waals).

Table S2. δ-SHTX-Hcr1f residue Arg13 non-covalent interchain with phospholipid, adjacent to VSD-IV helix S4.

Non-covalent interaction type[1]	δ-SHTX-Hcr1f Arg13 atoms	DPPC atoms	Energy contribution, kcal/mol
Hbond	NH1	O13	-8.1
Hbond	NH2	O13	-7.3
Ionic	NH2	O13	-6.707
Ionic	NH1	O13	-6.152
Ionic	NH1	O14	-2.119
Distance	NH2	P	-0.361
Distance	CG	C13	-0.316
Distance	CG	C15	-0.272
Distance	CZ	P	-0.171
Distance	CZ	C15	-0.16
Distance	NH2	NC3	-0.151

Distance	OD2	C15	-0.112
Distance	CZ	O14	-0.07
Distance	OD2	C13	-0.053
Distance	OD2	NC3	-0.017
Distance	OD1	C13	-0.016

[1] HBond - hydrogen bonds, Distance (hydrophobic, van der Waals).
